# The role of noninvasive and invasive diagnostic imaging techniques for detection of extra-cranial venous system anomalies and developmental variants

**DOI:** 10.1186/1741-7015-11-155

**Published:** 2013-06-27

**Authors:** Kresimir Dolic, Adnan H Siddiqui, Yuval Karmon, Karen Marr, Robert Zivadinov

**Affiliations:** 1 Buffalo Neuroimaging Analysis Center, Department of Neurology, School of Medicine and Biomedical Sciences, University at Buffalo, 100 High St., Buffalo, NY 14203, USA; 2 Department of Radiology, University Hospital Center Split, University of Split, Spinciceva 1, Split 21000, Croatia; 3 Department of Neurosurgery, School of Medicine and Biomedical Sciences, University at Buffalo, 100 High St., Buffalo, NY 14203, USA

**Keywords:** Multiple Sclerosis, CCSVI, Jugular Vein Reflux, Doppler Sonography, Magnetic Resonance Venography, Computed Tomography Venography, Catheter Venography, Intravascular Ultrasound, Plethismography, Multimodal Imaging

## Abstract

The extra-cranial venous system is complex and not well studied in comparison to the peripheral venous system. A newly proposed vascular condition, named chronic cerebrospinal venous insufficiency (CCSVI), described initially in patients with multiple sclerosis (MS) has triggered intense interest in better understanding of the role of extra-cranial venous anomalies and developmental variants. So far, there is no established diagnostic imaging modality, non-invasive or invasive, that can serve as the “gold standard” for detection of these venous anomalies. However, consensus guidelines and standardized imaging protocols are emerging. Most likely, a multimodal imaging approach will ultimately be the most comprehensive means for screening, diagnostic and monitoring purposes. Further research is needed to determine the spectrum of extra-cranial venous pathology and to compare the imaging findings with pathological examinations. The ability to define and reliably detect noninvasively these anomalies is an essential step toward establishing their incidence and prevalence. The role for these anomalies in causing significant hemodynamic consequences for the intra-cranial venous drainage in MS patients and other neurologic disorders, and in aging, remains unproven.

## Introduction

The venous system is a complex, low-pressure, freely communicating network of vessels, which contains 75% of the body’s circulating blood volume. The main function of the venous system is to return blood to the heart from the periphery and maintain cardiac output. Pathology in the peripheral venous system is frequently encountered and well-characterized as exemplified by varicose veins and deep vein thrombosis [1,2].

The extra-cranial venous system is complex as compared to the peripheral venous system, not well-examined and only partially understood [3,4]. It is a complex three-dimensional (3D) structure that is often asymmetric and represents significantly more variability than extra-cranial arterial anatomy. For example, unlike the carotid artery, the vascular wall of the internal jugular veins (IJVs) is much more flexible with a variable lumen diameter which can be influenced by postural change, respiration, cardiac function, hypovolemia and hydration status even by the pulsation of nearby arteries [5-10]. Even less is known about the main drainage routes of the spine, namely the azygous venous system and its pathophysiology. When performing imaging of the extra-cranial venous system, it is almost impossible to take all of the above factors into account, regardless of the imaging modality utilized. Moreover, because of the variant shapes and asymmetry of the IJVs, proper sizing is complex with common under- or over-estimation of the vessel diameter in regards presence of stenosis [11].

Currently, literature is relatively sparse in terms of investigation of the extra-cranial venous system as compared to the cerebrovascular arterial or peripheral venous systems. For almost two decades, uni- or bi-lateral jugular vein reflux (JVR) has been noted and related to several neurological disorders such as transient global amnesia, transient monocular blindness, cough headache and primary exertional headache [12-17]. However, only recently, a newly-proposed vascular condition, named chronic cerebrospinal venous insufficiency (CCSVI) [18], has generated an intense interest in better understanding of the role of extra-cranial venous anomalies and developmental variants, particularly in relation to the development of central nervous system (CNS) pathology [10,19-26]. CCSVI has been described as a vascular condition characterized by anomalies of the main extra-cranial cerebrospinal venous outflow routes that interfere with normal venous outflow in patients with multiple sclerosis (MS) [18,27,28].

The presence of the CCSVI implies a pathological condition for which diagnosis is based mainly on color Doppler Sonography (DS) findings in the extra-cranial (neck) and intra-cranial veins by assessing five venous hemodynamic (VH) criteria (with cutoff of ≥2 positive criteria used for diagnosis of CCSVI) [18,27]. The reliability of using DS in the diagnosis of CCSVI is questionable without proper training [29-31] and has been the focal point of recent statements from various societies [32,33].

Additional noninvasive modalities, such as magnetic resonance venography (MRV) [30,31,34-44] or computed tomography venography (CTV), may facilitate greater intra-cranial and extra-cranial vein examination, including that of the azygous vein in the chest, leading to an improved knowledge in this area, specifically, the anatomy of normal cerebrospinal venous outflow.

Although catheter venography (CV) is widely considered the “gold standard” for the assessment of vascular anomalies, including CCSVI [28,34,42,43,45-51], there is a lack of standard CV protocol or established guidelines for optimal diagnostic assessment of CCSVI diagnosis. There are significant differences between CV techniques and its interpretation among angiographers with no scientific evidence supporting a particular angiographic technique. Moreover, the rules implied in arterial imaging cannot be used for the imaging of extra-cranial veins.

### Venous anomalies vs. developmental variants

The venous system development through stages may be associated with a number of developmental variants that do not necessarily represent pathological findings [52-54]. It has been reported that the extra-cranial venous anomalies are likely to be truncal venous malformations [53] characterized by intra-luminal defects, (such as flaps, webs, septums, membranes and malformed valves) [18,31,45] or by extra-luminal anomalies represented by stenoses of the venous wall [18,28,31,45,46,48,49,51]. Pathological studies aimed to define the nature of these venous anomalies/developmental variants are limited and more investigations are needed [55,56]. Diaconu *et al*. examined the IJVs, the brachiocephalic veins and the azygos vein from 20 cadavers (10 control and 10 MS patients) and concluded that the anatomy of the extra-cranial venous system has significant variability, including a differing number of valves in different regions and variable characteristics of the valves [56]. Coen *et al*. examined specimens from the IJVs of MS patients who underwent surgical reconstruction of the IJV, specimens of the great saphenous vein used for surgical reconstruction and specimens from patients without MS [55]. They found that extra-cranial veins of MS patients showed focal thickenings of the wall associated with a higher expression of type III collagen in the adventitia. Further studies are needed to define extra-cranial venous anomalies/developmental variants that cause significant hemodynamic alterations in the drainage of intra-cranial venous system and to determine their incidence and prevalence in aging, MS and other CNS disorders.

### Controversy and debate that triggered need for standardization and development of imaging procedures

Although the CCSVI hypothesis has provoked great controversy and debate in the MS research community since it was first presented [20,23,24,57-61], it gained popularity among MS patients because of the postulated possibility of venous insufficiency correction using endovascular procedures [28]. So far, there have been several contradictory studies published [28,46,49,62-68] and verified scientific evidence supportive of a causative relationship between CCSVI and MS is lacking [10,69]. As with many promising, yet unproven therapies, many MS patients have undergone endovascular treatment for CCSVI [70-74]. Patients have undergone these endovascular procedures in either open-label or private care settings but largely in non-randomized, non-blinded and poorly controlled clinical settings [69]. Some of the central tensions of the CCSVI debate are related to the fact that the safety and efficacy of endovascular treatment have not been investigated and proven to be beneficial in randomized, controlled, blinded trials. So far there have been several case reports concerning patients who had serious side effects after angioplasty for CCSVI like IJV stent thrombosis requiring open thrombectomy, stent migration, aneurysmal vein dilatation, cranial nerves neuropathy, as well as reports of lethal cases [48,49,63,75]. Because patients with other neurologic diseases (OND) and healthy individuals may present with CCSVI, it is unclear whether the correction of CCSVI is necessary and whether it can lead to objectively measured improvements [76].

There is an increasing interest in imaging the extra-cranial venous system and great need for determination of the imaging “gold standard” for the detection of extra-cranial venous anomalies and developmental variants [76,77]. In our view, additional research and effort is needed until clear and uniform answers are found [76].

This article summarizes current knowledge regarding the advantages and disadvantages of both noninvasive and invasive imaging modalities for the detection of these extra-cranial venous anomalies and developmental variants that have been associated with CCSVI (Tables 1 and 2). This article also describes the need for standardization and development of guidelines.

**Table 1:** Advantages and disadvantages of noninvasive diagnostic methods for diagnosis of chronic cerebrospinal venous insufficiency

**Noninvasive diagnostic methods**	**Advantages**	**Disadvantages**
**Doppler sonography**[18,27,30-32,34,78-101]	- noninvasive	- no standardized guidelines
	- without ionizing radiation	- operator dependent
	- less expensive	- time consuming (60 to 120 minutes)
	- high resolution	- blinding procedures are challenging
	- real time information	- cannot perform global view of the veins (limited window)
	- sensitive to detect flow changes, intra- and extra-luminal abnormalities	- misidentification of the veins
	- ability to measure velocity	- influenced by hydration status
	- possible control of respiratory phases	
**Magnetic resonance venography**[10,30,31,34,42,43,102]	- noninvasive	- no real time information
	- without ionizing radiation	- cannot detect intra-luminal abnormalities
	- well established method	- low specificity of conventional MRV techniques
	- operator independent	- influenced by hydration status
	- less time consuming than DS	- azygos vein examination needs technical improvements due to important artifacts (breathing, heart movements)
	- provide global view of intra- and extra-cranial venous system	- underestimates the vascular caliber
	- easy to blind	- “snapshot” nature
	- ability to measure flow and velocity with advanced technique (phase contrast MRV)	
	- global view of collateral veins	
	- can be performed without contrast (pregnancy, allergy)	
**Computed tomography venography**[5,103,104]	- noninvasive	- ionizing radiation
	- less expensive and time consuming than MRV	- no real time information
	- better spatial resolution than MRV	- cannot detect intra-luminal abnormalities
	- global view of veins	- cannot be performed without contrast (allergy, toxicity)
	- lack of experience for extra-cranial venous system	- less contrast resolution than MRV
**Plethysmography**[105,106]	- noninvasive	- higher false-positive rate due to venous compression arising from incorrect patient positioning or the action of extrinsic masses
	- provides valuable information regarding the impact of reflux and obstruction on overall venous function	- low resolution
	- can monitor the dynamics of venous disease over time and evaluation of treatment outcomes	

Legend: DS, Doppler sonography; MRV, Magnetic Resonance Venography.

**Table 2:** Advantages and disadvantages of invasive diagnostic methods for diagnosis of chronic cerebrospinal venous insufficiency

**Invasive diagnostic methods**	**Advantages**	**Disadvantages**
**Catheter venography**[28,34,42,43,45-51]	- considered gold standard	- invasive method
	- real time information can be obtained by using contrast	- ionizing radiation
	- ability to measure pressure	- cannot be performed without contrast (allergy, toxicity)
	- provide “road map” for planning endovascular procedures	- operator dependent
	- can be complemented by use of more sophisticated criteria (time to empty contrast from vein or wasting of the balloon)	- time consuming (>45 minutes)
		- cannot detect intra-luminal abnormalities
		- no global view of veins and collaterals
		- no standardized definition of significant vein stenoses
**Intravascular ultrasound**[47,107,108]	- offers a 360° view of the vessel’s wall from the inside	- invasive method
	- can detect intra-luminal abnormalities	- lack of experience - no standardized protocols
	- easily accesses all parts of IJVs in comparison with DS	- ring down artifacts
	- provides more accurate assessment of vein stenosis and wall thickness than CV and DS	- geometric distortion - from imaging in an oblique plane
		- size of IVUS probe - limitation in the imaging of severe stenosis

Legend: CV, catheter venography; DS, Doppler Sonography; IVUS, intravascular ultrasound.

## Noninvasive imaging modalities

### Doppler sonography

#### Advantages

**Figure 1:**
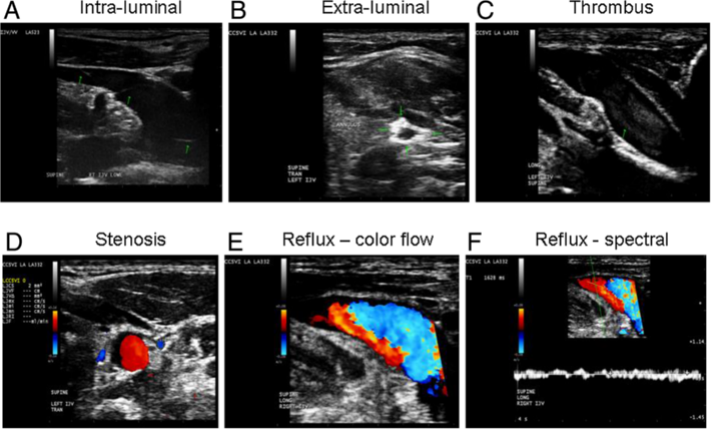
**Examples of chronic cerebrospinal venous insufficiency venous hemodynamic criteria on Doppler sonography. (A)** Flap anomalies noted in internal jugular vein (IJV) lumen; **(B)** annulus in the left IJV: circumferential thickened vein wall that is restricting the vein from fully expanding with respiratory or positional changes; **(C)** thrombus noted in IJV; **(D)** severe stenosis of left IJV: CSA measurement of ≤3 mm^2^; **(E** and **F)** Reflux/bidirectional flow directed towards the brain for a duration of >0.88 seconds in the right IJV in the supine position (**E** demonstrates reflux using color flow, while **F** demonstrates reflux using spectral analysis - waveform noted above baseline for more than 0.88 seconds).

DS is clinically the most useful technique for detecting, localizing and evaluating peripheral venous obstruction and venous valvular incompetence [33,109]. The sensitivity and specificity of venous DS for symptomatic proximal deep vein thrombosis exceeds 90% [110,111]. Spectral analysis of the DS signal is used to confirm the presence or absence of flow and indicates its direction and the patterns. Spectral analysis of DS signal and color DS are used to confirm the presence of reflux. It has the advantage among other diagnostic techniques of being noninvasive, providing high-resolution images with real time dynamic information, such as flow and velocity, showing intra-luminal (Figure 1A) as well as extra-luminal anomalies and developmental variants (Figure 1B) and being considerably less expensive than other noninvasive imaging techniques. DS imaging can also be readily applied in the follow-up period of subjects undergoing endovascular treatment because it can recognize the associated complications (residual stenosis, restenosis or venous thrombosis) (Figure 1C) [28,67,68].

Recent findings suggest that the majority of CCSVI pathology is confined to the intra-luminal portion of extra-cranial veins, which requires high-resolution B-mode imaging for the visualization of these anomalies [31,47]. The visible "stenoses" (Figure 1D) or extra-luminal venous anomalies most likely develop more frequently, merely with the progression of the disease or age [10].

**Figure 2:**
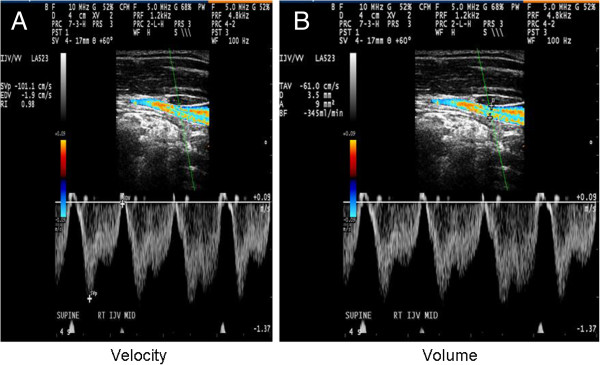
Example of velocity (A) and volume (B) measurement over four-second phase in internal jugular vein (IJV).

Because of the advantages of DS in detecting intra-luminal venous pathology, it was initially promoted as a method of choice for the screening of extra-cranial venous anomalies and developmental variants, indicative of CCSVI [18,27]. The diagnosis of CCSVI is both based on hemodynamic and imaging findings that utilize DS to study the deep cerebral veins, the IJVs and the vertebral veins (VVs) in both erect and supine positions. DS can also assess the hemodynamic consequences of outflow derangement while B-mode ultrasound detects structural venous intra-luminal anomalies (Figure 1E, F) [18,27,31,33,109,112]. Zamboni *et al*. created a set of five DS VH criteria by which MS patients were differentiated from healthy controls with 100% specificity and sensitivity [18,27] (Figure 1). However, in their original publication [18], they did not recommend exact technical procedures for the protocol application in either a research or routine clinical setting. The first attempt to define the standardized CCSVI scanning protocol was recently presented [98]. More recently, the International Society for the Neurovascular Disease (ISNVD) developed a more comprehensive consensus document that included the participation of more than 40 international experts in DS imaging. DS was proposed as a standardized screening tool for determining CCSVI status [33]. The protocol proposes the use of quantitative measures for the definition of functional anomalies, such as blood flow velocity and volume (Figure 2) that could be potentially more reliable in assessing the degree of venous outflow obstruction in the IJVs. It also refines originally proposed VH criteria. Even more recently, the European Society of Neurosonology and Cerebral Hemodynamics (ESNCH) expressed considerable concerns regarding the accuracy of the proposed criteria for CCSVI in MS [32], and proposed the central blinded DS reading as part of a recent multi-center Italian CoSMo study investigating the prevalence of CCSVI in MS patients, controls and patients with OND [113].

#### Disadvantages

**Figure 3:**
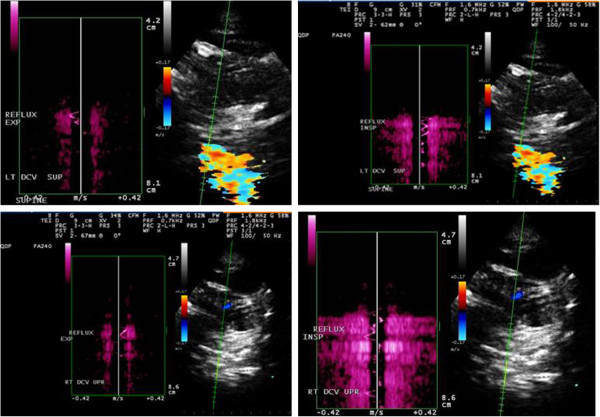
**Example of reflux in the deep cerebral veins using Quality Doppler Profile (QDP).** Doppler profile on opposite sides of baseline.

The main criticism of the recommended DS protocol is that its reproducibility depends on the training level and skills of the operator and it is not easy to be blinded and standardized in either a research or clinical setting [29-33,87]. Moreover, the value of the CCSVI VH criteria is controversial because they combine functional and structural intra- and extra-cranial venous anomalies/developmental variants in a single binary composite. Zamboni *et al*. used ≥2 abnormal DS VH criteria as a cutoff for CCSVI diagnosis classification [18,27]. The dichotomous variable construct of the CCSVI diagnosis, based on the arbitrary decision biased toward characteristics of the originally studied population and on the obtained results without further testing and validation datasets [18,27], may contribute to explaining major inconsistencies in the prevalent findings of CCSVI between different studies ranging from 0 to 100% [18,27,34,78-98,100,101,114]. The assessment of the second CCSVI criterion (reflux in deep cerebral veins) (Figure 3) is particularly controversial because the direction of the blood flow in veins connecting cortical with deep veins may vary considerably as a consequence of the physiologic inter-individual variation of the cerebral venous anatomy [30,32,33,87].

DS also has limits regarding extra-cranial vein characterization, since findings can be influenced by hydration status [10]. DS is a very time-consuming method and visualization of the central veins, particularly in the thorax and abdomen, is often limited and cannot give the global view of vein anatomy. Although it can detect extra-cranial collateral veins, which are probably associated with CCSVI, it is not technically feasible to follow the complete course of the collateral veins, which can be more easily visualized with use of MRV, CTV or CV [10]. Other pitfalls in DS imaging include the misidentification of veins. Additionally, overlying bone and muscle may prevent continuous imaging (cannot visualize suitably the confluence of the IJVs and the subclavian vein because clavicle commonly blocks direct visualization). Similarly, the cervical part of IJV and the jugular bulb cannot be visualized by DS because of the limited acoustic window resulting from the spine, mandible and skull [10,112,114].

#### Prevalence findings of CCSVI

So far, none of the recently published DS studies [30-32,34,78-101] have reproduced the originally reported CCSVI prevalence [18,27], regardless of the diagnostic DS method utilized. Even those DS studies which detected a significant difference for CCSVI diagnosis between MS patients and the controls, reported a substantially lower prevalence than was originally reported [30,31,83,88,90,92-94,98,99].

The largest cohort published to date of MS patients and controls with DS examined in a blinded manner reported prevalence rates of 56.1% in MS patients, 42.3% in those with OND, 38.1% in clinically isolated syndrome and 22.7% in healthy controls [98]. There have been numerous additional DS studies that showed significant differences in CCSVI prevalence between MS patients and the controls [30,31,78,83,88,90,92-95,99]. However, an even higher number of DS studies have failed to show prevalence differences in CCSVI between MS patients and controls [34,80-82,84-87,89,91,96,97,100],[101].

By using contrast-enhanced DS to assess cerebral circulation times (CCT) in MS patients and control subjects, Mancini *et al*. showed that MS patients had a significantly prolonged CCT and more frequent retrograde flow in IJVs [90].

#### Jugular vein reflux

Several studies have shown a relationship between IJV drainage anomalies, characterized by JVR and specific neurological diseases of undetermined etiology, such as transient global amnesia [14], transient monocular blindness [17], cough headache [13], primary exertional headache [16], idiopathic intra-cranial hypertension [115] along with a higher prevalence of white matter hyperintensities in older people [15]. JVR was also investigated in a large cohort of elderly subjects. An increased prevalence of JVR, dilated vessel lumen and slowed flow velocity in the left IJV, as well as decreased time-averaged mean velocity of bilateral IJV, was found in those over 70 years of age [116].

### Further considerations

The prevalence of CCSVI and JVR, as well as their relationship to clinical findings in CNS disorders, emphasizes the need for more quantitative and reproducible measures for the integration of morphological and functional anomalies. These include blood flow, as well as velocity and blood volume that could be potentially more reliable in assessing the degree of venous outflow obstruction in the IJVs and azygous vein (Figure 2). Contrast-enhanced exams can potentially increase the value of DS [90]. There is a need for training and use of standardized VH criteria for the diagnosis of CCSVI, as recently reported [33,98]. While the value of these VH criteria in detecting venous anomalies or developmental variants is uncertain [32], no other validated criteria have been proposed at this time. We hope that rapidly growing literature will contribute to the refinement of protocols and procedures to be utilized in the study of the extra-cranial venous system [113].

### Magnetic resonance venography

MRV is an often overlooked and underappreciated noninvasive and safe method for the evaluation of head and neck veins. Academic and clinical applications of MRV are relatively meager by comparison to CV or DS [117]. In relation to DS, the advantages are driven by MRV being a noninvasive technique, less time-consuming and less operator-dependent. MRV can also depict, easily and globally, the anatomy and morphology of the head, intra-and extra-cranial venous system. MRV is a comprehensive, noninvasive and relatively operator-independent technique which provides a 3D structural assessment of the intra and extra-cranial vasculature for the potential identification of stenosis and quantification of blood flow through major veins [41].

Recent studies have used MRV to assess differences between the MS population and controls with varying degrees of success. MRV has been tested against other imaging modalities, such as CV and DS, in detection of venous anomalies [30,31,34,42,43,102]. Wattjes *et al*. performed MRV in 20 MS patients and 20 age- and gender-matched controls and found no significant difference in the rate of venous anomalies [40]. The authors concluded that the anomalies in venous outflow had likely reflected normal developmental anatomic variants. Another study also reported no differences between 21 MS patients and 20 controls in relation to IJVs outflow and aqueductal cerebrospinal fluid flow using phase-contrast sequences and contrast-enhanced MRV [37]. Zivadinov *et al*. found no difference in morphological flow characteristics between MS patients and controls [44]. However, Dolic *et al*. found that progressive MS patients showed more morphological anomalies than those in relapsing stages of the disease [31]. Only one MRV study, so far, that included 19 MS patients and 20 healthy controls showed a significant difference in flow morphology of the IJVs between the two groups [36]. MS patients had greater flattening of the IJVs than healthy controls with no difference in collateral scores. The findings of these studies suggest that MRV morphologic information by itself may be insufficient to allow conclusions to be drawn about the presence of venous anomalies and their relationship to CCSVI in MS.

MRV is extremely useful in detecting collateral veins, which probably represent physiological variations of the venous system that may play a compensatory role when there are more venous extra-cranial anomalies present [30,31]. The extra-cranial venous collateral circulation probably represents a compensatory mechanism for impaired venous outflow, because it bypasses blocked veins and thereby reduces resistance to drainage [10]. The assessment of the possible prominence or collateralization of the extra-cranial veins in the neck by MRV is an important diagnostic step in examining the status of the venous system.

#### Time-of-flight

During the past decade, catheter-based digital subtraction angiography, as the preferred method for imaging of the intracranial venous anatomy, has been increasingly supplanted by MRV, usually performed with a two-dimensional time-of-flight (TOF) pulse sequence [118]. In the absence of better non-invasive techniques for the imaging of the dural venous sinuses, well-known and documented pitfalls associated with flow-sensitive MR techniques have been tolerated.

Furthermore, simple protocols that incorporate 2D-TOF acquisitions have already improved their accuracy for the diagnosis of deep venous thrombosis involving the femoral, popliteal or iliac veins [119]; however, experience with these techniques in the cervical veins is still limited. Thoracic central veins are largely inaccessible by DS, and MRV is an excellent technique for the assessment of axillary, jugular, subclavian, superior vena cava and pulmonary veins. TOF venography has the advantage of simplicity because no special pulse sequences are required and this technique is available on nearly every MRI system. TOF pulse sequences are spoiled gradient-echo or gradient echo acquisitions performed sequentially, that is, all phase encode steps are played out in a single slice before moving on to the next slice that results in much greater suppression of stationary tissue. It also has the advantage of avoiding the need for use of contrast agents and it remains the technique of choice in the evaluation of the pregnant patient with suspected dural sinus thrombosis. Furthermore, the accompanying conventional MR study is more sensitive in terms of the detection of cortical venous infarction than a CT [120]. Additionally, CTV always requires the use of intravenous contrast, while many non-contrast methods are available with MRV, making MRV the preferred technique in patients who also suffer from renal insufficiency or contrast allergy. CTV may also require two or more acquisitions to adequately capture contrast opacification of the veins, thereby increasing the radiation dose [103].

**Figure 4:**
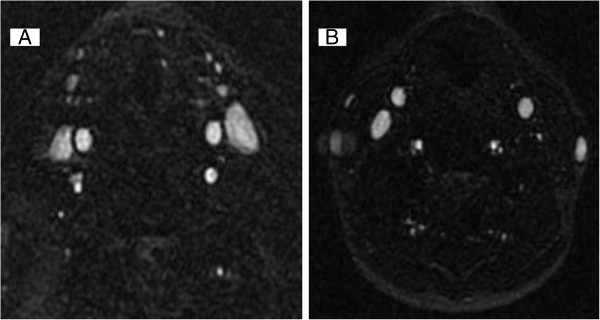
**Example of normal and abnormal flow in internal jugular vein on magnetic resonance venography.** Normal flow in both internal jugular veins **(A)** and abnormal flow in left internal jugular vein on axial 2D time-of-flight **(B)**.

The axial orientation of the acquisition allows for high in-plane resolution, which is ideal for cross sectional area (CSA) measurements of the veins. However, the TOF sequence is easily affected by motion artifacts, especially from the patient’s breathing, swallowing, snoring or head motion [38,41] (Figure 4). Relative insensitivity to in-plane flow is another limitation of the TOF technique. Regarding the direction of flow, the optimal acquisition plane lies orthogonal, which is inefficient from the standpoint of acquisition time and not always achievable. Although it has a higher spatial resolution 2D-TOF may overestimate stenosis in the setting of turbulent or slow flow [42].

All in all, standard conventional MRV techniques are more prone to artifacts than phase-contrast MRV and 3D-TOF angiography [10,44]. These techniques can potentially alleviate some of the usual MRV artifacts and provide more detailed flow information. One obvious improvement is to image at higher field strength, such as 3T, because this increases signal-to-noise ratio and better characterizes slow flow.

#### Phase contrast imaging

**Figure 5:**
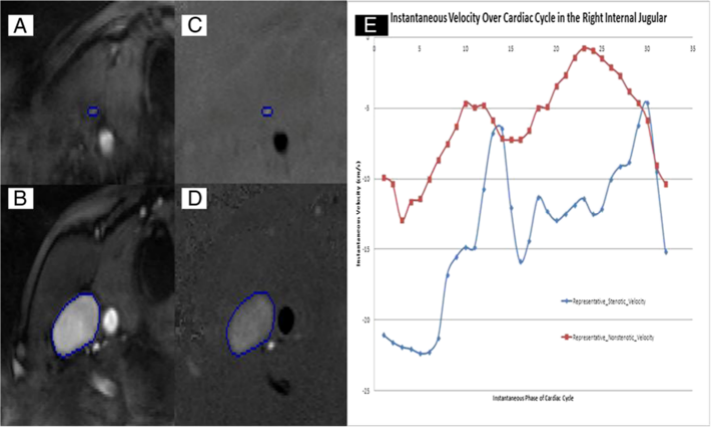
**Example of internal jugular vein pathology on cine phase**-**contrast MRI study.** The regions of interest (ROIs) outlined are the internal jugular veins. These ROIs were used to measure the flow through these vessels. An example showing the flow quantification magnitude image in stenotic **(A)** and normal IJV **(B)** and the flow quantification on phase images of the same IJVs **(C** and **D)**. Graph showing the differences in velocity between stenotic and non stenotic IJV **(E)**.

In contrast to TOF techniques, which rely mainly on flow-related enhancement for producing vascular images, phase-contrast MR angiography (PC-MRI) uses velocity-induced phase shifts imparted upon the moving spins to distinguish flowing blood from the surrounding stationary tissue, thus providing information regarding both anatomy and flow (Figure 5). The major advantage of PC-MRI angiography is excellent background suppression as well as quantitative determination of blood velocities. However, it requires long imaging times and a prior estimate of blood flow velocity. Furthermore, it may also be more sensitive to signal loss due to turbulence or intravoxel dephasing [121,122]. So far, to the best of our knowledge, there are only a few studies that used PC-MRI to quantify venous flow in MS patients. Sundström *et al*. studied the IJV flow normalized by the total arterial flow at the C2/C3 levels in 21 MS patients and 20 controls and found no statistically significant difference between the two [37]. On the other hand, Feng *et al*. characterized and compared the flow characteristics in a large cohort of the non-stenotic and stenotic MS patients and observed significantly reduced IJV flow in the stenotic group [41]. They concluded that a normalized total IJV flow of less than 50% of total arterial flow may be a potential biomarker for identifying significant stenoses in IJVs. Additionally, Haacke *et al*. showed that patients suffering from MS with structural venous anomalies on MRI exhibit an abnormal flow distribution of the IJVs [35]. In contrast to PC-MRI, Hartel *et al*. used very simple MRV protocol with T2FatSat and 2D-TOF sequences for the assessment of flow disturbances in IJVs and azygous vein [123]. They found that abnormal flow pattern in IJVs in MS patients is more common on the left side.

More studies are needed to validate the venous flow at the upper neck level on an adequate number of age- and gender-matched healthy controls with heterogeneous age groups.

#### Contrast-enhanced techniques

**Figure 6:**
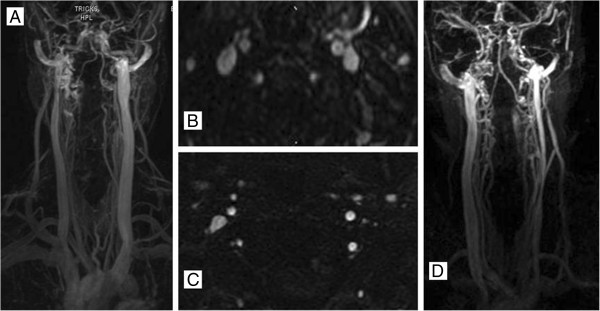
**Example of normal and abnormal flow morphology in internal jugular vein on magnetic resonance venography.** Normal **(A** and **B)** flow morphology in both and abnormal **(C** and **D)** flow in the left internal jugular vein on enhanced 3D time-resolved imaging of contrast kinetics (TRICKS).

Contrast-enhanced (CE) MRV, 3D time-resolved imaging of contrast kinetics (TRICKS) angiography is a noninvasive and safe method for the evaluation of head and neck veins, without the attendant risks of conventional angiography. It is preferred over TOF angiography because contrast medium reduces the T1 relaxation time of blood and virtually eliminates the effect of saturation [124,125] (Figure 6).

CE MRV is probably the most widely-used technique and is essentially identical to 3D CE MR angiography, employing a 3D-spoiled gradient-echo sequence in conjunction with a bolus of gadolinium-based contrast. Vascular contrast results from the T1-shortening effects of gadolinium on adjacent water protons and has relatively little dependence on inflow effects. In contrast to MRA, the limitation of CE MRV is that maximal contrast enhancement achieved in veins is typically lower than arteries because the contrast bolus is more dilute by the time it reaches the venous system [126]. To improve background suppression and emphasize vascular signal, fat saturation can be added to a 3D spoiled gradient-echo sequence with a small increase in acquisition time. 3D reconstruction of CE MRV data is somewhat less straightforward than MR angiography reconstruction since the vein/background contrast is lower and there is usually arterial as well as venous enhancement.

Veins can have variable MR imaging signal intensity due to entry slice phenomenon, in-plane flow, flow turbulence effects and can have variable enhancement. The maximum intensity projection (MIP) volumetric reconstructions of these sequences often underestimate the vascular caliber, especially when there are segments with decreased flow (velocity or volume) [120].

Disadvantages of CE MRV include the expense of the contrast agent, as well as contrast toxicity and patient discomfort in obtaining antecubital venous access. In the case of dural sinus thrombosis, however, confident early diagnosis of this common and treatable disease can dramatically reduce patient morbidity.

#### 4D flow imaging

Another promising MR technique is cine velocity-encoded phase-contrast 4D flow that may permit evaluation of not only anatomic stenoses but also their impact on venous waveforms. It is based on the principle that moving protons change phase in proportion to their velocity. By enabling a qualitative assessment of the presence and direction of collateral circulation, velocity-encoded cine MR imaging provides information about the presence and severity of obstruction. The technique has been most extensively used for the evaluation of patterns of blood flow in the thoracic aorta, including the characterization of abnormal flow patterns associated with pathologic disorders, such as ascending aortic aneurysm and dissection [127]. Recent studies have explored the use of 4D flow imaging for other areas of vascular anatomy and pathology, including intracranial arterial and venous blood flow [128]. With its detailed characterization of complex, dynamic blood-flow patterns and its ability to quantify flow, the technique could supplement both current noninvasive and invasive imaging of intra- and extra-cranial vascular pathologic disorders. The diagnostic and monitoring value of 4D flow imaging of venous flow anomalies, indicative of CCSVI, is currently lacking.

#### Further pitfalls and considerations

Finally, MRV suffers from its "snapshot" nature. An accurate depiction of these veins requires multiple views and maneuvers, such as inspiration and expiration, flexion and extension as well as rotation imaging of the neck. Its main disadvantages are the lack of MRV dynamism in real time, lower resolution compared with DS and CV (cannot evaluate intra-luminal pathology, such as the immobile valves, webs, septations, membranes and duplications) and it is affected by the nature of the veins themselves, which are prone to collapse under frequently encountered conditions, as opposed to arteries. MRV often detects spurious stenoses that are not confirmed by CV, especially in the lower parts of IJVs [42,123]. These stenoses may represent transient phasic narrowings (functional) or may result from diminished flow above true stenoses commonly located at the confluent region of the veins [30,31,102,123]. Additionally, it cannot satisfactorily evaluate the azygous and hemiazygous veins.

Unlike DS, with most MR scanners, data can only be collected in the supine position, although some scanners can do an upright scan as well. Niggemann *et al*. used positional MR imaging to describe the influence of positional changes on the cerebral venous outflow [129]. They found that IJV strictures are a common finding in healthy controls in the supine position without relevance in the erect position, which questions the validity of the DS VH criterion 5 (lack of collapse of the IJV in upright posture) for the diagnosis of CCSVI. It is obvious that this criterion (to study the change in flow in the IJVs from supine to sitting position) cannot be studied with the conventional MR system [130].

### Computed tomography venography

The development of spiral CT has greatly extended the range of venous evaluation. Previous reports have noted that CTV has a high sensitivity for depicting the intracerebral venous circulation as compared with digital subtraction angiography [103]. Advantages of CTV over CV include decreased cost, noninvasiveness and time to diagnosis. The CTV source images can also demonstrate parenchymal anomalies not detectable with CV and it has the ability to display images in rotating three-dimensional cine loops (as well as MRV), which provides a virtually limitless number of views from a single injection [104].

**Figure 7:**
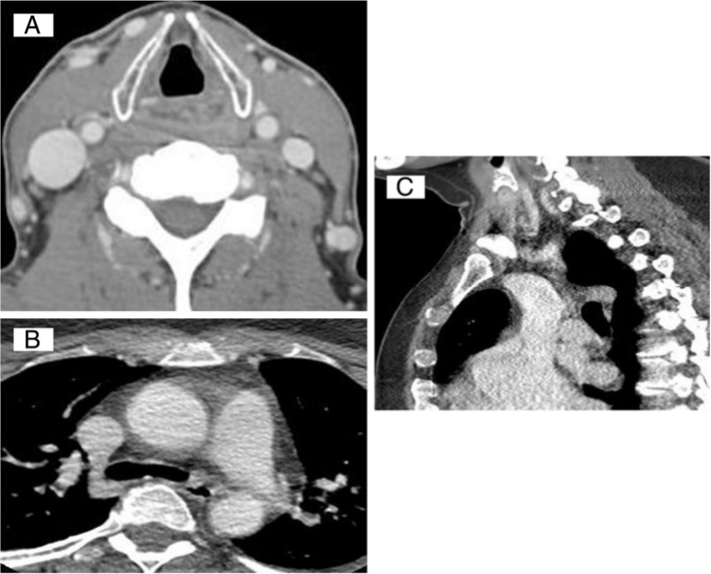
**Example of computerized tomography venography of internal jugular and azygos veins.** Axial **(A)** reconstructions of internal jugular veins. Axial and sagittal reconstructions of azygos vein **(B** and **C)** are shown, but their diagnostic value is questionable.

Further, CTV is superior to MRV in the identification of cerebral and extra-cranial veins and dural sinuses based on speed along with spatial resolution, and is at least equivalent in establishing the diagnosis of dural sinus thrombosis. It is also less expensive and less time consuming. Examination is very short; hence, the image quality is hardly impaired by patient motion, putting it as a first choice in critically ill patients [5]. Many patients who are not candidates for MRV by virtue of pacemakers, other MRI incompatible devices or claustrophobia can be examined with CTV. On the other hand, venous contrast-to-noise ratios are almost always higher with MRV. CTV also, like MRV, cannot evaluate intra-luminal vein pathology, such as the immobile valves, webs, septum, membranes and duplications. In relation to CCSVI, some of the main advantages of CTV may be related to venous multi-planar and global venous system evaluation, possibility of direct assessment of the azygos vein (morphology, caliber, course and possible calcifications) and use for therapeutic planning [10]. Nevertheless, there are no case–control CTV studies in MS patients. This is most likely due to the potential for radiation exposure to controls and need for use of a contrast agent. Our group gained preliminary experience by using CTV as part of a multimodal diagnostic approach in a currently on-going “Prospective Randomized Endovascular Therapy in MS (PREMiSe)” study (Figure 7).

## Invasive imaging modalities

### Catheter venography

CV is usually considered to be the “gold standard” for defining the degree of stenosis in blood vessels associated with altered blood flow [28,42,45,48]. However, it has been found to be less sensitive in revealing the exact nature of narrowed extra-cranial vein segments. Although CV is a luminogram, it brings little or no data regarding the vessel's intra-luminal structures, because of dense opacification of the lumen with contrast, which obliterates subtle intra-luminal structures. Although it is excellent in detecting larger intrusions, such as atheromas into the lumen, it has limited potential to detect lesions, such as intra-luminal valve malformations, septa and flaps [10,107]. Though it is possible to use very dilute contrast and cone-downed images at high rates of acquisition to pick up some of these intra-luminal features, they are generally harder to detect on CV using conventional acquisition parameters and contrast strengths. Therefore, in cases where only the intra-luminal venous anomalies or developmental variants are present, it is extremely difficult to measure the degree of flow obstruction by CV. In addition, malformed and/or reversed valve cusps can be crossed by the catheter and kept open artificially, thereby preventing the documentation of stenosis. Conversely, CV has several important advantages, including the ability to perform pressure gradient measurements as well as to provide a helpful “road map” for planning endovascular procedures [28,46,107]. However, its invasiveness, use of contrast agents and radiation exposure make it suboptimal as a routine screening tool in a clinical setting. It is also operator dependent, only AP projection views are routinely obtained and stenosis assessment may depend on the precise locations and rates of contrast injection.

One of the main criticisms of the CCSVI concept arose from the use of endovascular procedures to unblock potentially stenotic IJV and azygos veins in open-label fashion without previously establishing a) diagnostic imaging modalities and protocols that will serve as a “gold standard” for the detection and monitoring of these extra-cranial venous anomalies and b) safety as well as efficacy of the endovascular procedures in randomized, double-blinded, sham-controlled studies [10,28,46,49,62-66,69,71,131]. Furthermore, classification, existence and interpretation of venous anomalies are questionable, given the fact that the same can be found among healthy populations [40,85,88,98]. At this time, it remains unclear whether extra-cranial venous anomalies represent an acquired pathology or developmental variants. Future longitudinal studies need to elucidate these important questions.

The challenge at this moment, given the early stage of CCSVI related studies, is in defining the venous anomalies and developmental variants being detected with CV and the criteria being used to make subsequent treatment decisions. Additionally, there are lingering questions regarding the best vascular access. These questions include: Whether to use diluted or non-diluted contrast? Should these veins be evaluated irrespective of their diameter and anatomy of the venous network? What parameters should define pathological valve and other intra-luminal structures and should routine CV of these veins always be accompanied by intravascular ultrasound (IVUS) [48,108]?

**Figure 8:**
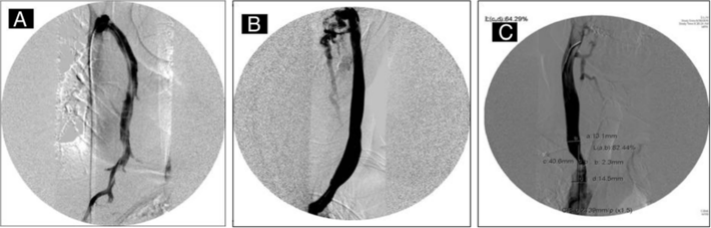
**Catheter venography of azygos and internal jugular veins.** Example of normal patent lumen of the azygos vein **(A)** and left internal jugular vein (IJV) **(B)**. Significant stenosis of the proximal right IJV **(C)**.

It is apparent that the invasive methods for the assessment of hemodynamic stenoses in the extra-cranial venous system, (mostly IJV and azygos veins), are not optimal. The first finding to consider when evaluating a patient for CCSVI is the degree of narrowing within the vein as seen on CV and the decision as to what constitutes a significant stenosis. The IJV is often not a circular object; often being oval or complex. Thus, determination of the diameter of the vein by CV is often arbitrary and, therefore, it under- or over-estimates the proper size of the balloon for the angioplasty [108]. The concept of a significant obstruction being when the vessel has been reduced to 50% of its diameter, (which corresponds to a 75% reduction in CSA), is derived mainly from observations in the arterial system. However, these criteria may not be applicable in the venous system because there are some fundamental differences. One potential issue is that the IJV can vary significantly in both size and symmetry with various factors, including hydration status, cardiac output, respiratory excursions as well as head position that can account for some of the noted variability [26]. Using DS at the level of the cricoid cartilage, Lin *et al*. found that the normal venous diameter ranged from 9.1 mm to 10.2 mm but that a small IJV (5 mm in diameter) can be seen in 13.5% of subjects on the right side and in 10.6% on the left side [132]. In light of the high pressures necessary to dilate the stenosis, proper sizing is crucial to avoid injury to the vein by over dilation- or early recurrent stenosis by under-dilatation (Figure 8). More sophisticated categorical criteria (ranging from grade 1 to grade 4) have been recently proposed [46] but they need to be tested and validated. Further, there is the concern that an intra-luminal anomaly, such as septae, may easily be displaced out of the way by an inflated balloon but upon deflation fall right back in its original position and continue to functionally obstruct flow.

CV can only show the collaterals that drain the specific vein being injected without the possibility of showing the global extra-cranial venous system at once, that is, as with MRV or CTV [10,43]. The display of extra-cranial venous structures can be improved with additional injected contrast medium, more selective catheterization and additional projections.

Although a number of open-label studies evaluated extra-cranial veins in MS patients and showed a high prevalence of venous anomalies [28,43,45-51,107,133], there are no data available comparing CV findings in MS patients and age- and sex-matched controls. The availability of such studies is essential in determining the potential prevalent differences between venous anomalies or developmental variants, indicative of CCSVI and their general distribution in the healthy pediatric and adult population with respect to age and gender.

#### Future considerations

CV can be complemented by use of more sophisticated criteria such as time to empty contrast from the vein or wasting of the balloon across a stenosis [134]. Further, with the ability to perform pressure gradient measurements before and after the endovascular procedures it can indirectly give the information about hemodynamic significance of venous obstruction [28].

### Intravascular sonography

Intravascular sonography (IVUS) is an endoluminal CV-based US technique that offers a tomographic, 360° view of the vessel’s wall from the inside. It also allows more complete and accurate assessment than is possible with the use of CV examination. Therefore, IVUS imaging may reflect truly the size of stenotic lesions. It provides cross-sectional, *in vivo* visualization and the demonstration of the motility of small intra-luminal structures, which cannot be optimally revealed by traditional diagnostic methods [135].

The most common indications for IVUS have been in the evaluation and treatment of arterial disease. Its excellent resolution compared with angiography has contributed to the understanding of the pathophysiology and enhanced diagnosis of coronary artery disease achieving new milestones in interventional cardiology [136-138]. IVUS has been shown to provide a more accurate assessment of vessel circumference and cross-sectional area and thus, is useful in detecting critical stenoses. Analysis of the vessel dimensions allows a more accurate selection of balloon size, thus reducing the risk of injury and providing a more effective angioplasty [139,140]. Abnormal valves characterized by highly echogenic irregular thickening, poor mobility, bulging cusps, as well as septum, and webs are more easily seen by IVUS because they are highly echogenic. It has been shown that such venous pathology in the iliac vein is unrecognized by CV and is well visualized by IVUS [141].

**Figure 9:**
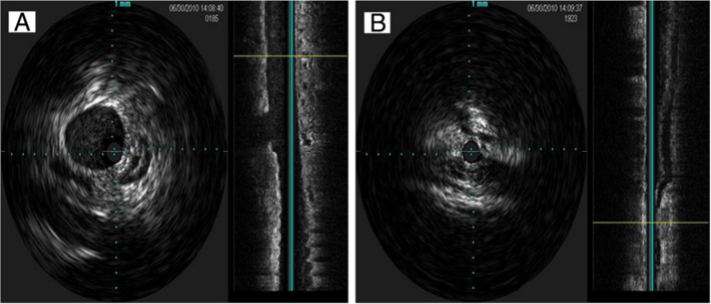
**Example of intravascular ultrasound in the internal jugular vein.** Normal patent lumen **(A)** and stenotic lumen **(B)** with fibrotic wall.

Although diagnostic experience is growing with the use of IVUS for investigation of both intra- and extra-cranial arteries [142], there is limited literature regarding its use for the exploration of venous vasculature in general, as well as specifically in relation to the investigation of venous anomalies and developmental variants indicative of CCSVI [47,107,108] (Figure 9). It is our experience that IVUS is more accurate in the detection of intra-luminal venous anomalies in IJVs and azygos vein, more accurate in measurement of stenosis and wall thickness and allows for the exploration of pulsatility in the veins [134]. The exploration of IJV valves is particularly well-seen on IVUS. Additionally, thrombus and dissections are readily seen on IVUS [108]. IVUS can also show the degrees of echogenicity, both of the vessel wall and of the intra-luminal thrombi, which may indicate varying degrees of wall thickness and may correlate with the age of the thrombosis, an important aspect of the vessel pathology that is not possible to be determined with CV [143].

In a recent study that included 45 MS patients, Scalise *et al*. found that CV was significantly inferior to DS and IVUS in detecting intra-luminal anomalies. IJV CSA was under-estimated by DS compared to IVUS [107]. In another study, Lugli *et al*. retrospectively examined 167 consecutive MS patients who presented ≥2 positive DS VH criteria [47]. In 37% of the cases there was no correlation between the preoperative DS assessment and the CV findings. In the event of incongruity between the two exams, IVUS was performed and confirmed DS findings in 42% of cases and CV results in 58%. Karmon *et al*. have explored the prevalence of extra-cranial venous anomalies in IJVs and azygous using CV as well as IVUS in 30 MS patients who showed ≥2 positive DS VH criteria [132]. CV was considered abnormal when ≥50% lumen restriction was detected, whereas IVUS was considered abnormal when ≥50% restriction of the lumen or intra-luminal defects or reduced pulsatility was found. Venous anomalies detected by IVUS were observed in 85% of azygous vein, 50% of right IJVs and 83.3% of left IJVs, whereas CV showed stenosis of ≥50% in 50% of azygous vein, 55% of right IJVs and 72% of left IJVs. CV sensitivity for detecting IVUS anomalies was 52.9%, 73.3% and 80% for the azygous vein, left IJV and right IJV, respectively. This study showed that the IVUS assessment of IJVs and azygous vein can detect higher rates of venous anomalies than CV and that provides a diagnostic advantage over the "gold-standard" CV in detecting extra-cranial venous anomalies and developmental variants indicative of CCSVI.

#### Advantages

The advantages of IVUS compared with DS, among others, include the sonographic penetration from within the vessel by excluding extra-vascular soft tissues. It also assesses blood vessels not easily accessible by conventional DS, such as the lower part of the IJV (behind the clavicle), upper part of the IJV, intracranial sinuses and azygos vein. Additionally, it provides an image with a greater resolution of both lumen and wall (with additional 3D features), providing better vessel wall information. IVUS is superior in identifying intra-luminal venous anomalies/development variants compared to CV [107,108,134]. Moreover, CV is incapable of monitoring respiratory pulsatility which involves periods with reduced vessel diameter that can be investigated with IVUS. While values for stenosis definition used for CV (≥50%) rely on a ratio between the stenotic segment diameter and a pre-(non) stenotic vein, which is more variable, the IVUS definition is more strict (a lumen that embraces the IVUS probe for a critical stenosis) and does not refer to a non-stenotic segment [134]. It remains unclear at what level and with what criteria is there a significant hemodynamic effect of stenosis by either modality. Venous stenosis is currently measured using arterial criteria, which are clearly not optimal. The hemodynamics of venous flow remain a major area of investigation and better understanding will likely lead to a revision of stenosis criteria.

#### Disadvantages

Ring-down artifacts produced by acoustic oscillations in the piezoelectric transducer that obscures the near field, results in an acoustic catheter size larger than its physical size and may adversely affect IVUS images [144]. Geometric distortion can result from imaging in an oblique plane (not perpendicular to the long axis of the vessel) [145]. Furthermore, visible distortion of the image can be due to another important artifact, "non-uniform rotational distortion", which arises from uneven drag on the drive cable of the mechanical style catheters, resulting in cyclical oscillations in rotational speed. The physical size of IVUS catheters (currently approximately 1.0 mm) constitutes an important limitation in the imaging of severe stenoses [146]. Further, depending on the probe there is a finite limit to IVUS resolution which rapidly degrades beyond this particular radius typically 10 to 12 mm. In summary, the frequency of the transducer, gain settings, depth of penetration and focal depth are some of the factors that affect the sensitivity of the IVUS imaging.

#### Further considerations

Further studies are needed to validate the role of IVUS in depicting extra-cranial venous anomalies and developmental variants, indicative of CCSVI. Protocol optimization and standardization are needed to make this imaging method more widely used. Preliminary IVUS studies that investigated extra-cranial venous anomalies and developmental variants have been extremely important in better understanding these structures [47,107,108,134].

### Plethysmography

Plethysmography is the only existing practical noninvasive modality for global physiologic evaluation of extremity veins. As such, it provides valuable information regarding the impact of reflux and obstruction on overall venous function and can provide a measure of calf muscle pump function (strain-gauge plethysmography) [147,148]. The identification and assessment of venous obstruction by plethysmography is based on an estimation of these two parameters: venous capacitance and venous resistance.

The use of plethysmography as a complementary modality to DS is reasonable for quantification of reflux or obstruction, for monitoring the dynamics of venous disease over time and for the evaluation of treatment outcomes. Despite their value in the anatomical localization of disease, imaging modalities such as DS and CV cannot assess the global severity of reflux or obstruction. Moreover, the use of strain-gauge or air-plethysmography to diagnose venous thrombosis in the lower extremities has been well- documented [148,149]. By inflating a cuff on the thigh, the constriction of veins causes the venous volume to rise. When the cuff is released, the sensor detects rapid venous runoff and a return to the resting blood volume. If thrombosis is present, the plethysmography will detect a delay in the emptying process. Unfortunately, as with their invasive counterparts, most of the non-invasive tests display the fundamental dichotomy of providing either anatomic or hemodynamic information. Plethysmography can be prone to a higher false-positive rate due to venous compression arising from incorrect patient positioning or the action of extrinsic masses. It is also a time-consuming method [149].

Quite recently, plethysmography has been used to measure endothelial function as well as the vascular response to vasoactive agents [150]. The technique is rarely used in the cervical region. Zamboni *et al*. recently showed that cervical plethysmography is much less prone to operator error compared to DS and has great potential to be used as an inexpensive diagnostic tool for demonstrating extra-cranial venous anomalies and development variants [105]. Further, Begss *et al*. conducted a study with 40 controls and 44 CCSVI patients who underwent cervical plethysmography, which involved placing a strain-gauge collar around their necks and tipping them from the upright (90°) to supine position (0°) in a chair and demonstrated that hemodynamics of the extra-cranial venous system are greatly altered in CCSVI patients [106].

#### Further considerations

Apart from these early studies, little work has been done on the application of cervical plethysmography in the detection of extra-cranial venous anomalies and developmental variants. Further research is needed in identifying cutoff values, the reproducibility of the test along with assessing intra- and inter-observer variability. This methodology also shows great potential in monitoring postoperative patients after restorative endovascular procedures.

### Multimodal imaging approach

**Figure 10:**
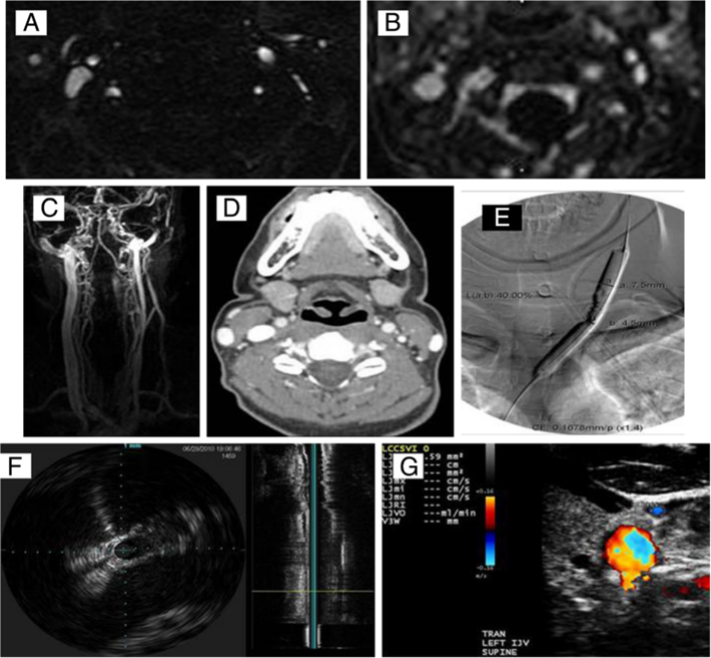
**Example of multimodality imaging of extra**-**cranial neck veins in the PREMiSe study (Prospective Randomized Endovascular Therapy in MS).** Axial 2D time-of-flight **(A)**, enhanced 3D-time resolved imaging of contrast kinetics **(B** and **C)**, Doppler sonography **(D)**, catheter venography **(E)**, intravascular sonography **(F)** and axial computed tomography venography (**G**) all showing venous abnormality of the left internal jugular vein (narrowing).

The dramatic difference in prevalent findings between different studies using non-invasive and invasive imaging techniques (ranging from 0% to 100%) emphasizes the urgent need for the use of a multimodal imaging approach for better understanding of the venous anomalies and developmental variants being considered in CCSVI [10]. In a number of recent studies, noninvasive and invasive imaging techniques were applied and compared [18,27,30,31,34,42,43,47],[50,81,82,102,107,134]. The findings of these studies are extremely important to understand the true prevalence of CCSVI and the comparison of invasive vs. noninvasive imaging findings is especially important in this endeavor. It is emerging that the prevalence of venous anomalies and developmental variants, indicative of CCSVI is even higher, when investigated with sophisticated invasive imaging techniques [47,107,108,134]. Based on these recent findings, a multi-modal approach is recommended to determine whether CCSVI exists as a clinical entity and not as an anatomic variant, and to what extent it is present in various healthy and disease groups as well as MS subtypes (Figure 10). The introduction of more quantitative criteria to describe extra-cranial venous structural and hemodynamic functional impairment in future multimodal approach studies will be a significant improvement compared to the current binary CCSVI diagnosis.

## Conclusions

The use of noninvasive methods, such as DS, to confirm the diagnosis of CCSVI presently remains controversial. A consensus on DS protocols to ensure appropriate quality control for the determination of venous anomalies and developmental variants, indicative of CCSVI is essential [32,33,113]. Although a number of authors have proposed use of MRV as an alternative noninvasive diagnostic approach, no consensus currently exists. Thus, at present, the true prevalence of CCSVI in MS patients versus controls has not been adequately assessed.

Diagnostic studies in diseased and control populations using invasive imaging techniques, such as CV and IVUS, to detect venous anomalies and developmental variants indicative of CCSVI are essential to determine their true prevalence.

Because of the complexity and variability of the extra-cranial venous system, it is almost impossible to take all of the factors mentioned above into account, regardless of the imaging modality used. Each noninvasive and invasive imaging modality has its own inherent advantages and disadvantages (Tables 1 and 2). Most likely, only multimodal imaging will eventually become the reliable screening, diagnostic and monitoring tool for the assessment of the extra-cranial venous system.

Further research is needed to determine the spectrum of extra-cranial venous anomalies and developmental variants and to compare findings against pathological examinations [55,56]. Undoubtedly, the attention being focused on CCSVI has significantly contributed to the vast surge in research on the extra-cranial venous system.

Unfortunately, as a consequence of uncritical use of endovascular procedures, an increasing number of adverse events have been reported after angioplastic procedures for CCSVI. The ability to diagnose CCSVI noninvasively will be an essential step toward better understanding of its importance in general population and disease states.

## Abbreviations

CCSVI: Chronic cerebrospinal venous insufficiency; CCT: Cerebral circulation time; CE: Contrast-enhanced; CNS: Central nervous system; CSA: Cross sectional area; CTV: Computed tomography venography; CV: Catheter venography; DS: Doppler sonography; IJV: Internal jugular vein; IVUS: Intravascular ultrasound; JVR: Jugular vein reflux; MIP: Maximal intensity projection; MRA: Magnetic resonance angiography; MRV: Magnetic resonance venography; MS: Multiple sclerosis; PC: Phase contrast; PREMiSe: Prospective Randomized Endovascular Therapy in Multiple Sclerosis; TOF: Time of flight; VH: Venous hemodynamic; VV: Vertebral veins; 3D: Three dimensional; 4D: Four dimensional.

## Competing interests

Kresimir Dolic, Yuval Karmon and Karen Marr have no competing interests to disclose.

Dr. Siddiqui has received research grants from the National Institutes of Health (co-investigator: NINDS 1R01NS064592-01A1, Hemodynamic induction of pathologic remodeling leading to intracranial aneurysms) and the University at Buffalo (Research Development Award); holds financial interests in Hotspur, Intratech Medical, StimSox, and Valor Medical; serves as a consultant to Codman & Shurtleff, Inc., Concentric Medical, ev3/Covidien Vascular Therapies, GuidePoint Global Consulting, Penumbra and Stryker Neurovascular; belongs to the speakers’ bureaus of Codman & Shurtleff, Inc. and Genentech; serves on National Steering Committee for Penumbra, Inc., 3D Separator Trial; serves on an advisory board for Codman & Shurtleff; and has received honoraria from American Association of Neurological Surgeons’ courses, Genentech, Neocure Group LLC, Annual Peripheral Angioplasty and All That Jazz Course and from Abbott Vascular and Codman & Shurtleff, Inc. for training other neurointerventionists in carotid stenting and for training physicians in endovascular stenting for aneurysms. Dr. Siddiqui receives no consulting salary arrangements. All consulting is per project and/or per hour.

Dr. Zivadinov received financial support for research activities from Biogen Idec, Teva Pharmaceutical and Teva Neuroscience, EMD Serono, Genzyme-Sanofi, Novartis, Greatbatch, Bracco and Questcor. He also received personal compensation from Teva Pharmaceutical, Biogen Idec, Novartis, Genzyme-Sanofi, EMD Serono, Bayer, Novartis and General Electric for speaking and consultant services.

## Authors’ contributions

KD and RZ designed as well as drafted the article, while AHS, YK and KM revised it critically for important intellectual content. All authors approved the final version of the manuscript.

## Pre-publication history

The pre-publication history for this paper can be accessed here:

http://www.biomedcentral.com/1741-7015/11/155/prepub
